# Heterogeneity in mantle carbon content from CO_2_-undersaturated basalts

**DOI:** 10.1038/ncomms14062

**Published:** 2017-01-13

**Authors:** M. Le Voyer, K.A. Kelley, E. Cottrell, E.H. Hauri

**Affiliations:** 1 Carnegie Institution of Washington, Department of Terrestrial Magnetism, 5241 Broad Branch Road NW, Washington, District Of Columbia 20015-1304, USA; 2 Graduate School of Oceanography, University of Rhode Island, Narragansett Bay Campus, Narragansett Rhode Island 02882, USA; 3 Smithsonian Institution, National Museum of Natural History, PO Box 37012, MRC 119, Washington, District Of Columbia 20013-7012, USA

## Abstract

The amount of carbon present in Earth's mantle affects the dynamics of melting, volcanic eruption style and the evolution of Earth's atmosphere via planetary outgassing. Mantle carbon concentrations are difficult to quantify because most magmas are strongly degassed upon eruption. Here we report undegassed carbon concentrations from a new set of olivine-hosted melt inclusions from the Mid-Atlantic Ridge. We use the correlations of CO_2_ with trace elements to define an average carbon abundance for the upper mantle. Our results indicate that the upper mantle carbon content is highly heterogeneous, varying by almost two orders of magnitude globally, with the potential to produce large geographic variations in melt fraction below the volatile-free solidus. Such heterogeneity will manifest as variations in the depths at which melt becomes interconnected and detectable, the CO_2_ fluxes at mid-ocean ridges, the depth of the lithosphere-asthenosphere boundary, and mantle conductivity.

## Abstract (web summary)

Melting dynamics are affected by the amount of carbon in the Earth's mantle. Le Voyer *et al*. report undegassed carbon concentrations from olivine-hosted melt inclusions from the Mid-Atlantic Ridge finding that carbon content varies by two orders of magnitude thus introducing heterogeneity into the upper mantle.

The carbon content of undegassed mid-oceanic ridge basalts (MORB) and of the upper mantle has been an ongoing debate for several decades^1^,^2^,^3^,^4^,^5^,^6^,^7^,^8^,^9^. Carbon is a volatile element that plays a key role in major geodynamical processes such as mantle melting and volcanic degassing. The amount of carbon present in the mantle will affect the onset of deep melting, the geophysical properties of the mantle, as well as long-term climate change when CO_2_ is released into the atmosphere^1^. Because of its very low solubility^10^, magmatic degassing depletes carbon in the melt during ascent and eruption, which prevents direct measurements of the original carbon content of most basaltic melts formed in equilibrium with the mantle source. Previous studies have used indirect approaches to correct for degassing, such as isotopic fractionation models, vesicle size distribution or the composition of the gas trapped in vesicles^2^,^3^,^5^. Our knowledge of mantle carbon is best constrained by direct measurements of only two undegassed samples: the popping rock 2πD43 (ref. 2) and the Siqueiros melt inclusions^4^. Together these two samples show correlations between CO_2_ content and non-volatile trace elements (such as Nb) that directly constrain the amount of carbon in the upper mantle^2^,^4^, and thus both samples have served as references for CO_2_ fluxes due to volcanism and its effect on long-term climate changes^11^. Yet, the two most recent studies of these samples^2^,^4^ show that their CO_2_/Nb ratios differ by more than a factor of two, suggest that the mantle CO_2_/Nb ratio is either variable^2^ or constant^4^, and propose upper mantle CO_2_ abundances and mid-ocean ridge CO_2_ fluxes that vary by a factor of four. Another study reports unusually high CO_2_ contents in melt inclusions from the Juan de Fuca ridge^12^. However the decoupling between their CO_2_ and Nb contents indicates that part of the CO_2_ was lost through degassing before entrapment^12^. A recent study^13^ compiled most CO_2_ measurements from published MORB glasses and melt inclusions, along with new data from 15 ultradepleted MORB glasses that are undersaturated in CO_2_ and do not contain any vesicles. Because of this, the authors argue that they are mostly undegassed^13^. These undersaturated MORB glasses have average CO_2_/Rb, CO_2_/Ba and CO_2_/Nb ratios that are close to those of the Siqueiros melt inclusions, also ultradepleted^4^. Studies of additional undegassed samples are critical to understanding whether these differences are due to sampling bias or real geologic variability.

Here we report the CO_2_ contents of an independent set of olivine-hosted melt inclusions from the equatorial Mid-Atlantic Ridge (MAR). Using correlations between CO_2_ and highly incompatible elements, we show that these melt inclusions represent another rare occurrence of undegassed MORB, and we discuss the implications of our results with respect to the carbon content of the mantle and the carbon flux from the global ridge system.

## Results

### Description of the sample

We analysed the major, trace and volatile elements of Fo_86–90_ olivine-hosted melt inclusions from the glassy rim of a pillow basalt from the equatorial MAR. Sample EN061-5D-3A was dredged on-axis by R/V *Endeavor* in 1981 (5.185° S, 11.517° W) (refs 14, 15, 16). The host glass is a depleted MORB with La/Sm_N_=0.5, similar to the average depleted MORB composition (D-MORB, La/Sm_N_=0.5 (refs 17, 18)). The matrix glasses contain 0.118±0.004 wt% H_2_O, 206±14 p.p.m. CO_2_, 102±5 p.p.m. F, 1,014±66 p.p.m. S and 17.8±2.0 p.p.m. Cl (uncertainty is 2 s.d. on replicate analyses from 15 glass chips), which is typical for a depleted MORB erupted at ∼3,300 m below sea level. The matrix glasses are saturated to slightly oversaturated with respect to a CO_2_-rich vapour phase at the pressure of eruption. Their estimated pressure of vapour saturation is P_sat_=444±30 bar (ref. 10).

### Description of the melt inclusions

From this sample we analysed 23 melt inclusions for major, trace and volatile element content; all of the melt inclusions are free of shrinkage bubbles. They are glassy, tholeiitic basalts with MgO contents slightly higher than their host glasses. All but three of the melt inclusions have undergone <3% post entrapment crystallization (PEC), and so although we refer herein to their PEC-corrected chemistry, the correction is inconsequential to our results (see Methods). Incompatible minor and trace element contents range from highly depleted to compositions identical to those of the matrix glass.

### Trace element composition of the melt inclusions

**Figure 1:**
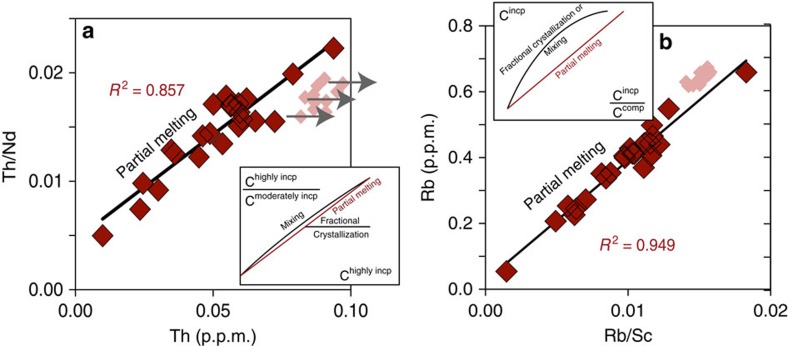
Variation in trace element composition of the equatorial MAR melt inclusons. (**a**) Th/Nd ratio as a function of Th content (p.p.m.) for the equatorial melt inclusions (red diamonds) and matrix glasses (pink diamonds). (**b**) Rb content (p.p.m.) as a function of Rb/Sc ratio for the equatorial MAR melt inclusions and matrix glasses.

We used the high-resolution trace element data set to assess which process is responsible for the large range and co-variation of the highly incompatible element contents in the equatorial Atlantic melt inclusions^19^,^20^,^21^,^22^,^23^. In a plot of C^H^/C^M^ versus C^M^, where C^H^ and C^M^ are the concentrations of a highly incompatible trace element such as Rb or Th and a moderately incompatible element such as Nd, partial melting is expressed as a straight line with a slope >1, while fractional crystallization would produce an almost horizontal line, and mixing would produce a curve^19^. In Fig. 1a, we see that the equatorial MAR melt inclusions plot on a straight line that is not horizontal, ruling out fractional crystallization as a main relationship explaining the range in composition of these melt inclusions. The small range in the major element contents of the melt inclusions (Supplementary Table 1) support this conclusion as well. Similarly, in a plot of C^H^ versus C^H^/C^C^, where C^C^ is the concentration of a compatible element such as Sc, mixing and crystallization would produce hyperbolic curves, while partial melting would generate a straight line^19^. In Fig. 1b, the straight line defined by the equatorial Atlantic melt inclusions confirms that they are related by variable degrees of melting. However we cannot rule out mixing as a possible cause for the large range in trace element compositions. The range in degree of melting calculated from the major element compositions (11–23%, using Na_(8)_ following ref. 24) is too small to account for the range in incompatible elements observed in the equatorial Atlantic melt inclusions (for example, Rb contents vary from 0.05 to 0.66, a 12-fold increase) using a simple batch melting model of a DMM-type mantle source^25^. The range in incompatible elements can be easily reproduced by modelling compositions of incremental melts produced during fractional melting, using a melt fraction varying from 0.7 to 1.0% (ref. 26). The range in incompatible trace element compositions could also reflect variable amounts of mixing between very low degree incremental melts early in the melting process (highly enriched in incompatible trace elements, such as the carbon-rich melts formed at depth beneath oceanic ridges^1^), and aggregated melts produced later in the melting process by ∼14% of melting. The incomplete pooling of melts is similar to the process described for the Famous segment^27^, where some of the melt inclusions trapped in olivines record compositions systematically more depleted than the matrix glasses. Alternatively, the large variation in trace elements could reflect a variable source composition, that is, a heterogeneous source containing a trace-element depleted source and a trace-element enriched source with up to ∼10-fold enrichment of the most incompatible trace elements compared to the depleted source. However, the compositions of the matrix glasses do not represent an average of the compositions of the melt inclusions, because the matrix glasses are most likely melt batches that are more enriched than the incremental melts preserved as melt inclusions. The absence of melt inclusions that record compositions more enriched than the matrix glass could reflect the fact that melt inclusions preferentially sample high degree melts^27^. Another hypothesis is that the matrix glasses are early-formed melts that mixed before cooling and olivine crystallization, while the melt inclusions are late-formed melts that are available in an unmixed form to be trapped in olivine. We favour the scenario of mixing of incremental melt batches produced from a single source at variable depths and variable degree of melting (that is, a deep melting event above the dry carbonated peridotite solidus, that produces very low degree melts, highly enriched in carbon and other trace elements, and another, more shallower melting event, above the dry peridotite solidus, that produces higher degree melts of the same source previously depleted by the first deep melting event^1^).

### Volatile element composition of the melt inclusions

**Figure 2:**
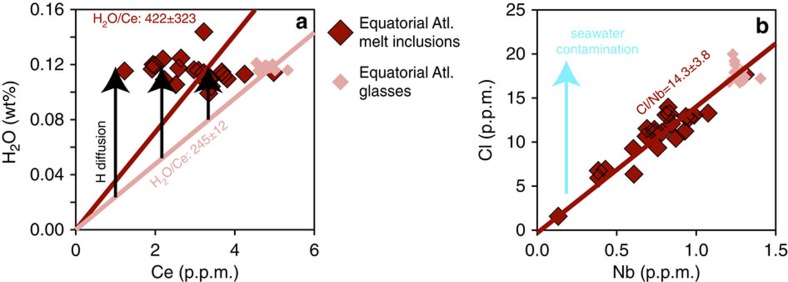
Behaviour of H_2_O and Cl in the equatorial MAR melt inclusions. (**a**) Variation in H_2_O content (wt%) as a function of Ce content (p.p.m.) for the equatorial MAR melt inclusions and matrix glasses. (**b**) Variation in Cl content (p.p.m.) as a function of Nb content (p.p.m.) for the equatorial melt inclusions and matrix glasses.

The melt inclusions contain 68–719 p.p.m. CO_2_, 52–90 p.p.m. F, 779–1,087 p.p.m. S, 1.6–17.7 p.p.m. Cl and 0.10–0.14 wt% H_2_O. Compared to the matrix glasses, which have suffered from strong CO_2_ degassing typical of most MORB glasses, the saturation pressures of the melt inclusions reflect minimum entrapment depths down to 4 km below the sea floor (*P*_sat_=150–1,500 bar). Because we do not observe shrinkage bubbles in the melt inclusions, the measured contents for the other volatiles, particularly CO_2_, represent those of the melts at the time they were trapped in olivine. The large range in volatile content is consistent with the range in other incompatible trace elements found in the melt inclusions. In Fig. 2a, we see that the equatorial MAR melt inclusions span a large range of Ce contents, similarly to other incompatible trace elements. However, H_2_O, which partitions similarly to Ce (ref. 28) only spans a very limited range of concentration (0.10–0.14 wt%), with an average H_2_O content in the melt inclusions identical to that of the matrix glass (0.118±0.004 wt%). At the depth of eruption (∼3,300 m.bs.l.), water degassing is only minimal (the equilibrium vapour phase contains <0.3 mol% of H_2_O); thus H_2_O/Ce should not be affected by degassing. Because most melt inclusions are depleted in trace elements compared to the matrix glasses, they should record lower H_2_O contents as well. The similar H_2_O contents recorded in the melt inclusions and the matrix glass illustrates a late-stage H_2_O enrichment in these depleted melt inclusions, by diffusive re-equilibration of hydrogen^29^ between the H_2_O-depleted melt inclusions and the H_2_O-rich matrix glasses, most likely during storage and differentiation in a shallow magma reservoir before eruption. If we assume that all melt inclusions had a H_2_O/Ce ratio similar to those of the matrix glasses (245±12), we can reconstruct the initial H_2_O content of the melt inclusions (0.03–0.12 wt% H_2_O, average of 0.07 wt% H_2_O). The amount of H_2_O gain through diffusion varies from none up to 2.8 times compared to the estimated initial H_2_O contents. We report in Fig. 2b the Cl content of the melt inclusions and matrix glasses as a function of their Nb content. Unlike most MORB samples, which tend to show variable amounts of seawater-derived Cl contamination, and therefore a variable Cl/Nb, the equatorial Atlantic melt inclusions have a constant Cl/Nb ratio of 14.3±3.8. A similar value is recorded in the matrix glasses. This indicates that the equatorial melt inclusions as well as matrix glasses did not suffer from significant alteration or assimilation of hydrothermal material^30^.

### Correlation between CO_2_ and other trace elements

**Figure 3:**
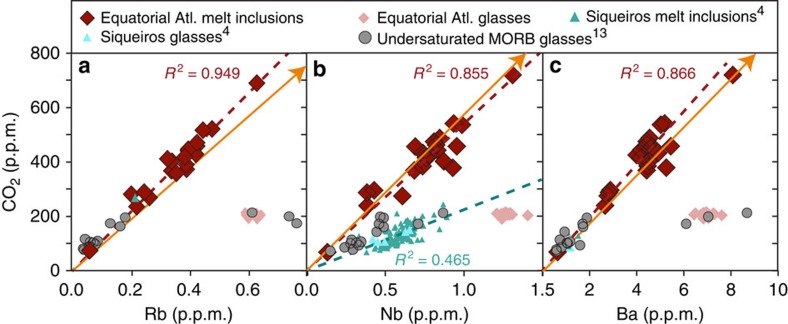
Variations in CO_2_ content as a function of highly incompatible trace elements. The CO_2_ content of the equatorial MAR melt inclusions and matrix glasses is represented as a function of Rb (**a**), Nb (**b**) and Ba (**c**), for, and compared to the Siqueiros melt inclusions and matrix glasses^4^, the popping rock (outside of the scale, represented by the arrow, CO_2_ and Nb (ref. 2); Rb and Ba (ref. 31)), and the undersaturated ultradepleted MORB glasses^13^. The single Siqueiros Rb point represents an average calculated using the Rb and Ba correlation found in Siqueiros melt inclusions^48^, applied to the maximum Ba measured in Siqueiros melt inclusions^4^. *R*^2^ values refer to the linear regressions (dashed lines) through each set of samples.

**Figure 4:**
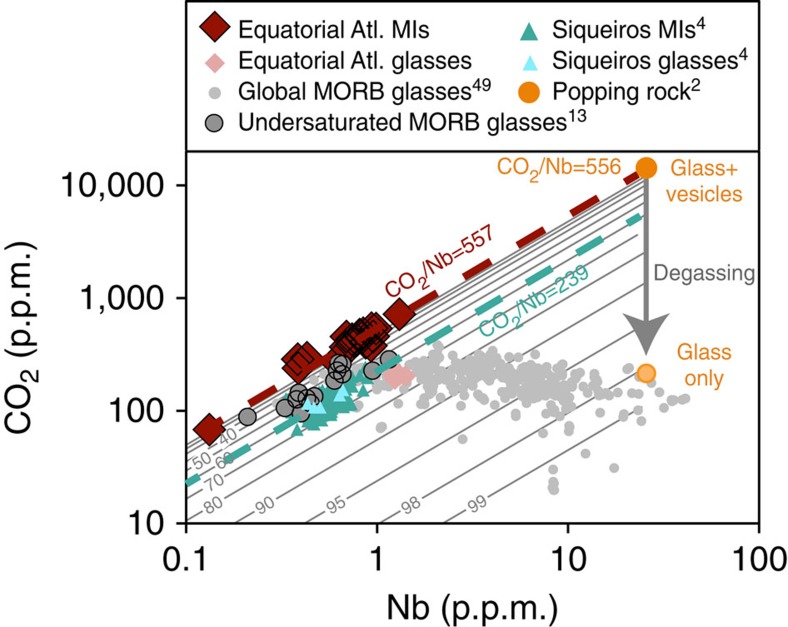
Variation in CO_2_ content as a function of Nb content. Red dashed line: average CO_2_/Nb in the equatorial MAR melt inclusions. The Siqueiros melt inclusions^4^ (green dashed line: average CO_2_/Nb) and matrix glasses^4^, the popping rock^2^ and the undersaturated ultradepleted MORB glasses^13^ are also plotted for comparison. The matrix glasses from both Siqueiros and equatorial MAR samples plot consistently with the global MORB glass database^49^, affected by variable amounts of carbon degassing (thin grey lines, graduated in % of CO_2_ loss, assuming an initial undegassed CO_2_/Nb of 557).

The CO_2_ content of the melt inclusions strongly correlates with other highly incompatible elements such as Ba, Rb and Nb, which indicates that these melt inclusions did not lose their initial carbon through degassing (Fig. 3). Note that the CO_2_ content of the equatorial melt inclusions also strongly correlates with their Cl content (CO_2_/Cl=39±4, 2 s.d., linear regression of *R*^2^=0.912). For comparison, the CO_2_/Cl ratio of Siqueiros melt inclusions is 77±26 (ref. 4), and the CO_2_/Cl ratio of popping rock is 46 (ref. 2). However the CO_2_/Cl ratio was not used below because, although the equatorial melt inclusions do not show any signs of seawater contamination, most MORB samples do. Therefore the use of the CO_2_/Cl ratio as a source proxy cannot be generalized to the global scale, as most MORB samples are strongly contaminated with seawater-derived Cl (ref. 30). The average CO_2_/Rb and CO_2_/Ba ratios of the equatorial Atlantic melt inclusions (1,105±104 and 97±10, Table 1) are only slightly higher than those for the popping rock 2πD43, and very similar to those for Siqueiros melt inclusions (Table 1, Fig. 3). The average CO_2_/Nb ratio of the equatorial MAR melt inclusions (557±79) is identical to the popping rock 2πD43, but approximately two times higher than the Siqueiros melt inclusions (Table 1; Figs 3 and 4). The equatorial MAR melt inclusions have a CO_2_/Rb and CO_2_/Ba ratios that are indistinguishable from those of the undersaturated MORB glasses^13^, and a CO_2_/Nb ratio that is 50% higher. The similarity between the undersaturated MORB glasses^13^ and our undegassed melt inclusions reinforces the conclusion that these undersaturated MORB glasses are mostly undegassed as well, although three out of their 15 glasses have lower CO_2_ content for a given Rb, Ba or Nb and might be degassed (Fig. 3).

## Discussion

Carbon is present as carbonate in the uppermost mantle under the oxidizing conditions relevant for the formation of MORB (refs 4, 31). Because very small extents of melting will efficiently remove carbonate minerals from the source, further melting will only dilute carbon in the pooled magma. The CO_2_-Nb correlation found in the Siqueiros melt inclusions indicates that C partitions similarly to Nb during melting and crystallization^4^. However, a recent experimental study found C to be slightly more incompatible than Nb, closer to Ba and Rb, and thus CO_2_/Nb is not a perfect canonical ratio at low degrees of melting^6^. Observations from a global compilation on MORB glasses also suggest that Ba is the best proxy for C to model the CO_2_ content of MORB and of the suboceanic mantle^13^. Nonetheless, at the degrees of melting typical of MORB generation, nearly all of the CO_2_, Rb, Ba and Nb from the mantle source are contained in the MORB melt, and thus CO_2_/Ba, CO_2_/Rb and CO_2_/Nb are all indicative of MORB source composition^6^. The correlations found between CO_2_ contents and highly incompatible trace element contents in the equatorial MAR melt inclusions (Fig. 3) confirm this observation. In the following, we use all three ratios in combination in order to assess the mantle CO_2_ content at the global scale.

Although it is unlikely that carbon is homogenously distributed in the upper mantle^7^, an average CO_2_ content is useful for many planet-scale geochemical and geophysical models. The average trace element composition of the equatorial MAR melt inclusions from this study is representative of the uppermost oceanic mantle away from any hotspot influence^17^. Similarly, the radiogenic isotope composition of the host glass^14^ is representative of average depleted MORB mantle (DMM). Thus, the values for CO_2_/Rb, CO_2_/Ba and CO_2_/Nb ratios measured in the equatorial MAR melt inclusions should be representative of the DMM as well, as opposed to Siqueiros melt inclusions^4^ and the ultradepleted MORB glasses^13^, which are highly depleted, and to the Popping Rock^2^, which is highly enriched. We used a compilation of Rb, Ba and Nb contents for global mantle averages from the literature (DMM values^25^,^32^ and DMM calculated from global MORB averages^17^,^18^,^33^,^34^,^35^, assuming that Rb, Ba and Nb are completely incompatible, and using an average melting degree of 10%) together with the CO_2_/Rb, CO_2_/Ba and CO_2_/Nb ratios from the equatorial MAR melt inclusions (Table 1). We obtain a global average CO_2_ in the mantle source of 137±54 p.p.m. CO_2_ (1 s.d. over the range in Rb, Ba and Nb from the literature estimates), equivalent to 37.4±14.7 p.p.m. C. The result is identical within error regardless of which ratio is used (CO_2_/Ba, CO_2_/Rb, or CO_2_/Nb). This CO_2_ abundance is within the range of previously determined abundances for the DMM from MORB of the Northern Atlantic province (∼175 p.p.m. (ref. 2), using an average CO_2_/Nb of 530, a MORB Nb content of 3.31 p.p.m., and assuming an average degree of melting of 10%), that determined from global MORB compilations (60–183 p.p.m. (ref. 13)), and lies between the estimates from the Siqueiros melt inclusions (72±19 p.p.m. (ref. 4), or 36 p.p.m. (ref. 32)) and the popping rock 2πD43 (∼300 p.p.m. (ref. 5)). Our value is twice as high as the average CO_2_ content of the mantle estimated from vesicle size distribution (66–78 p.p.m. (ref. 3)). The discrepancies between models show that these values are highly sensitive to which value of Rb, Ba or Nb, and which melting model is used^13^.

Assuming that our estimate of 137±54 p.p.m. CO_2_ is representative of the average DMM CO_2_ content, we calculate an average CO_2_ flux from ridges of 1.8±0.7 × 10^12^ mol yr^−1^, using a MORB flux of 21 km^3^ yr^−1^ (ref. 36), and an average degree of melting of 10%. The uncertainty on this estimate is conservative, as it takes into account the range in estimates of the Ba, Rb and Nb concentrations in DMM. This CO_2_ flux corresponds to an average ^3^He flux released from ridges of 802±316 mol yr^−1^, using a constant CO_2_/^3^He ratio for MORB of 2.2±0.7 × 10^9^ (refs 8, 9). The calculated CO_2_ flux is in very good agreement with previous estimates based on popping rock 2πD43 (2.3 × 10^12^ mol yr^−1^ (ref. 2)), and is twice as high as estimated from Siqueiros melt inclusions (9.3±2.8 × 10^11^ mol yr^−1^ (ref. 4)). Our CO_2_ flux is higher than the global estimates from vesicularity (6.5±1.8 to 8.7±2.8 × 10^11^ mol yr^−1^ (ref. 3)), and is slightly lower than those from a global MORB glass compilation (2.8±0.4 × 10^12^ mol yr^−1^ (ref. 13)). Normalized by the total length of the ridge system (60,864 km (ref. 17)), this corresponds to an average CO_2_ flux of 2.9±1.1 × 10^7^ mol yr^−1^ km^−1^. However, the CO_2_ flux from each segment of the global mid-ocean ridge system may vary as a function of magma flux and mantle source CO_2_ content. In the case of the ridge segment where sample EN061-5D-3 A was dredged, the local spreading rate of 3.26 mm yr^−1^ (ref. 17) and crustal thickness of 5 km (ref. 14) translate to a local magma flux of 0.0168, km^3^ yr^−1^, a local CO_2_ flux of 1.4±0.6 × 10^9^ mol yr^−1^, and a local ^3^He flux of 0.64±0.25 mol yr^−1^. Normalized by the length of the ridge segment (segment MAR179, 103 km long^17^), the local CO_2_ flux corresponds to an average flux of 1.4±0.5 × 10^7^ mol yr^−1^ km^−1^, which is half the average global flux. Thus, the differences observed between local and global CO_2_ fluxes illustrate geographical variations of at least a factor of two in the CO_2_ flux from ridges, controlled by variations in mantle carbon concentration and magma flux. This observation agrees with independent estimates based on MORB vesicularity^3^.

The equatorial MAR melt inclusions, an example of depleted MORB, have ratios of CO_2_/Ba, CO_2_/Rb and CO_2_/Nb very similar to the popping rock 2πD43, which is a highly enriched MORB (Fig. 3, Table 1). This observation demonstrates that these ratios are not simple functions of the amount of trace element enrichment or depletion in MORB; however, the limited range in these ratios, even taking Siqueiros into account, shows that absolute mantle CO_2_ abundances will scale with mantle Ba, Rb and Nb abundances. In order to successfully capture the global range of the upper mantle CO_2_ content, we use the global Rb, Ba and Nb variations from the literature, together with the CO_2_/Rb, CO_2_/Ba and CO_2_/Nb ratios from the equatorial MAR melt inclusions (Table 1). We selected estimates of Rb, Ba and Nb contents in both depleted MORB sources (D-DMM) and enriched MORB sources (E-DMM) (refs 17, 32), and obtain a range in DMM CO_2_ content of 20−1,200 p.p.m., equivalent to 5.5−327 p.p.m. C. This range, reflecting the full spectrum of depleted-to-enriched MORB sources, is wider than previously reported and covers almost two orders of magnitude, including the low^4^,^13^,^25^ and the high^2^ mantle CO_2_ estimates. This range is in good agreement with the global range estimated independently from vesicle size distribution (27–999 p.p.m. CO_2_ (ref. 3)). Our high CO_2_ end-member is higher than anything previously reported, and indicates that enriched mantle sources could contain much more carbon than previously suggested. This wide range in CO_2_ content shows the extent of carbon heterogeneity that is present in the mantle, and demonstrates that mantle source composition is an important contributor to the geographical variations in ocean ridge CO_2_ fluxes discussed above.

In a procedure similar to that for CO_2_, we provide estimates for the H_2_O, Cl and F content of the DMM. We do not apply this approach to S because the EN061-5D-3Ag are sulfide-saturated^37^; therefore, their S content is a direct function of the FeO content of the melt and cannot be linked to the S content of the mantle source. Water from the equatorial melt inclusions suffered from diffusive re-equilibration with the host glass, through H diffusion. Therefore we used the H_2_O content of the matrix glasses instead. We used a compilation of global averages for the mantle Ce, Nb and Zr contents from the literature (DMM values^25^,^32^ and DMM calculated from global MORB averages^17^,^18^,^33^,^34^,^35^), together with the H_2_O/Ce ratio from the equatorial matrix glasses (244±12, 1 s.d.), and the Cl/Nb and F/Zr ratios from the equatorial melt inclusions (Table 1). We obtained a global average of H_2_O, Cl and F contents in the mantle source of 238±68 p.p.m., 4.1±1.2 p.p.m. and 22±5 p.p.m., respectively. These values are higher than the DMM values of 142±85 p.p.m. H_2_O, 1.0±0.5 p.p.m. Cl and 16±3 p.p.m. F estimated from the Siqueiros melt inclusions^4^ as well as the DMM values of 0.5 p.p.m. Cl and 11 p.p.m. F (ref. 25).

**Figure 5:**
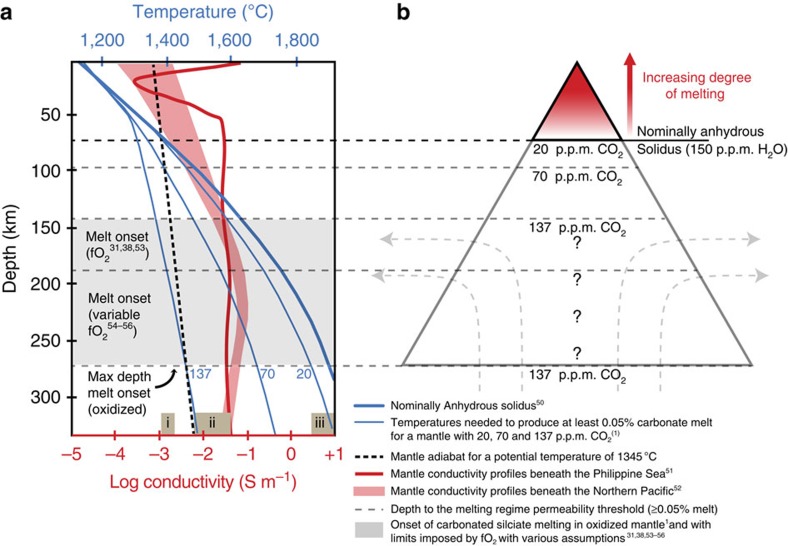
Simplified model of mantle melting. (**a**) Variation of melting temperatures with depth as a function of mantle CO_2_ content (blue curves)^2^,^50^, and electrical conductivity versus depth (red curves) for the upper mantle beneath the Philippine Sea^51^ (thin red curve) and north Pacific^52^ (broad red curve). Shaded boxes at the base of the plot represent the range of laboratory electrical conductivity measurements for dry olivine (i), olivine containing 150 p.p.m. H_2_O (ii) and melt (iii) (refs 30, 50). (**b**) Schematic cross-section of the permeable melting regime beneath a mid-ocean ridge based on **a**, using the same vertical scale, a mantle potential temperature of 1,345 °C and a permeability threshold of 0.05%. At depths below the nominally anhydrous solidus, melting is dominated by small fractions of carbonate and carbonated silicate melt. Shaded grey zones indicate possible, depths for carbonated silicate melt initiation depending on the oxygen fugacity (*f*O_2_) of the mantle^53^,^54^,^55^,^56^. Onset at the carbonated silicate solidus^1^ assumes that mantle *f*O_2_ is high enough to stabilize carbonate over diamond at 275 km. If the mantle is more reducing, melting will instead initiate at the *f*O_2_-dependent diamond to carbonate transition. Melt initiation between 140 and 180 km encompasses whole rock Fe^3+^/ΣFe ratios from 0.035 to 0.05 based on the continental xenolith record^38^—depths that are also consistent with a range of MORB Fe^3+^/ΣFe ratio, Fe^3+^ bulk partition coefficients, and primitive mantle Fe_2_O_3_ contents^19^ and the assumption that 37p.p.m. C has the power to reduce Fe^3+^/ΣFe by >1% as it oxidizes to carbonate. Because the subsolidus Fe^3+^/ΣFe ratio of the mantle and the depth of metal saturation are uncertain, the depth of melt initiation is also uncertain.

Our results on the CO_2_ content of the DMM have important implications for the geophysical detection of melt in the upper mantle and the origin of the asthenosphere. Provided that oxygen fugacity is high enough to stabilize carbonate^38^, carbonate melting will begin wherever the mantle temperature exceeds the carbonated mantle solidus, producing a melt fraction that is a function of the amount of CO_2_ in the mantle^1^. At the MORB mantle CO_2_ contents that we have constrained, this melt fraction will be vanishingly small. However, carbonate melts can become interconnected at very low melt fractions, as small as 0.05% (ref. 39), and thus there exists a melt fraction threshold below which carbonate melts cannot be extracted from the mantle and would not be detectable by geophysical methods. This threshold melt fraction can be used to define the effective base of the melting regime beneath ridges as well as the effective depth of melting. The seismic low-velocity zone beneath oceanic plates^40^ and the electrical conductivity structure of the upper mantle^41^ are both thought to be due to the presence of melt beneath the lithosphere. In particular, carbonate melts are highly conductive, much more so than hydrated mantle or silicate melts^42^. Using petrologic estimates for the reduction in melting temperature as a function of CO_2_ content^1^, a mantle potential temperature of 1,345 ° C and an interconnection threshold of 0.05% in melt fraction^39^, we show that the regional variations in upper mantle CO_2_ that we have documented here predict large variations in the depth to the effective base of the melting regime beneath ridges, which should correlate with geochemical trace element signatures of depletion and enrichment in MORB (Fig. 5). An upper mantle source with a CO_2_ abundance of 20 p.p.m. would produce 0.005% melt, which is an order of magnitude below the threshold for interconnection^39^ and would, in areas of trace element depletion, predict an absence of melt and low electrical conductivity at depths deeper than the nominally anhydrous mantle solidus (85 km (ref. 1)). A less depleted MORB source with 70 p.p.m. CO_2_, such as the source for Siqueiros MORB, would produce enough carbonated silicate melt to establish an interconnected (and thus conductive) network of melt at depths of ∼95 km and above. An average MORB source with 137 p.p.m. CO_2_ would produce an interconnected network of carbonated silicate melt throughout the entire upper mantle, limited at its base only by redox freezing where carbonate is converted to diamond^1^,^38^ (Fig. 5). Given sufficient depth resolution of electrical conductivity measurements, it may be possible to use geophysical measurements to determine the depth to the effective base of the carbonated melting regime in areas of trace element depletion, and the depth to the carbonate-diamond transition in areas of trace element enrichment. Given that the carbonate-diamond transition is dependent on oxygen fugacity^1^,^38^, variations in the depth of this redox boundary—if resolved—could be indicative of lateral variations in the oxygen fugacity of the mantle.

## Methods

### Sample preparation

We selected olivine grains that contained fully entrapped melt inclusions, with no cracks or links to the outside glass. We mounted the olivine grains in epoxy and polished them using SiC papers in order to expose the melt inclusions. After polishing, we removed the grains from epoxy using a soldering iron, pressed them into an indium mount, and polished the indium mounts using first diamond paste, then 1/3 μm alumina paste. We washed the mounts using alcohol and water, then stored them for >48 h in a vacuum oven at 70 ° C, before applying a gold coat.

### Volatile elements

We first analysed the volatile element compositions (H_2_O, CO_2_, F, Cl and S) of the matrix glass and the melt inclusions using the NanoSIMS Cameca 50L at the Department of Terrestrial Magnetism, Carnegie Institution, following the procedure described in ref. 43. We used a 12–13 nA, Cs^+^ primary beam to presputter the sample using a 30 × 30 μm^2^ raster, then performed the analysis using a 10 × 10 μm^2^ raster, and collected data on the central 3.8 × 3.8 μm^2^ of the crater, measuring 5 blocks of 10 analyses each. We assessed blank under these conditions by replicate measurements on Suprasil 3,002 glass and synthetic forsterite, which yield values of 0.2 p.p.m. CO_2_, 2 p.p.m. H_2_O and <0.05 p.p.m. F, Cl and S. Typical analytical error (2 s.d. over *n* analytical cycles) are <3% rel. for all volatiles. We assessed uncertainties by measuring basaltic glass ALV519-4-1 every 10 analyses, which yield uncertainties of 7% rel. for CO_2_ and <5% rel. for all other volatiles (2 s.d. over 50 analyses during a 4-day long analytical session). Supplementary Table 1 shows the long-term uncertainties as 2 s.d. external reproducibility of repeated measurements in four basaltic standards.

### Major elements

After measuring volatile elements, we slightly polished the samples using 1/3 μm alumina powder in order to remove the gold coat, then we applied a carbon coat. We measured the major element compositions of the host olivines, the matrix glasses and the melt inclusions using a JEOL electron microprobe at the Geophysical Laboratory, Carnegie Institution, using the following conditions: 15 kV accelerating voltage, 30 nA beam, spot mode (for the olivine) or beam defocused to 10 μm diameter (for the glasses). We processed the olivine analyses for matrix correction using the set of absorption coefficients from ref. 44. Supplementary Table 2 shows replicate measurements in basaltic glass standard VE-32. Supplementary Table 3 shows the major element compositions of the olivines.

### Trace elements

Finally, we analysed the concentrations of 40 trace elements in the melt inclusions and the matrix glasses by Laser-Ablation Inductively Coupled Mass Spectrometry at the Department of Terrestrial Magnetism, Carnegie institution, following methods adapted from refs 45, 46. We used a Photon Machines UV laser coupled with a Thermo iCapQ quadrupole ICP-MS. We ran the analyses using 100% energy output, 20 Hz repeat rate and 50 μm spot size. We normalized the data to ^29^Si as the internal standard. We used a set of 11 mafic glass standards (BIR-1g, BCR-2g, BHVO-2g, GSC-1g, GSD-1g, GSA-1g, BM90-21g, GOR132-g, GOR128-g, KL2-g and ML3B-g) to perform the calibration (linear regressions with *r*^2^>0.995). We assessed uncertainties and analytical drift using repeated measurement of basaltic glass standard VE-32, measured every 10 analyses of unknowns (Supplementary Table 4). We measured each sample three times, and combined accuracy and reproducibility on sample analyses is <10% (2 RSD) on average for all elements, except for Cs and U, whose low contents were close to or below detection levels.

### Post-entrapment olivine crystallization

We assessed the major, volatiles and trace element compositions of the melt inclusions for post-entrapment olivine crystallization (PEC). We used a Fe^3+^/Fe_t_ of 0.16 (average value measured in local MORB glasses^37^ identical to the global average MORB value^31^), together with the Fe-Mg partition coefficient between olivine and silicate melt^47^. We corrected the melts by adding olivine back to the melt using increments of 0.1%. All melt inclusions indicated an amount of PEC of ≤3% of olivine, with the exception of three melt inclusions that indicated PEC of 5, 7 and 9% of olivine. Supplementary Table 5 shows the PEC-corrected compositions of the melt inclusions and matrix glasses. Supplementary Table 6 shows the raw compositions of the melt inclusions and matrix glasses before PEC correction. Note that the main results of this study do not depend on the PEC correction, as both the use of the PEC-corrected compositions and the uncorrected compositions would yield similar conclusions.

### Data availability

The authors declare that all data generated during this study are included in this published article (and its Supplementary Information files).

## Additional information

**How to cite this article:** Le Voyer, M. *et al*. Heterogeneity in mantle carbon content from CO_2_-undersaturated basalts. *Nat. Commun.* **8,** 14062 doi: 10.1038/ncomms14062 (2017).

**Publisher's note**: Springer Nature remains neutral with regard to jurisdictional claims in published maps and institutional affiliations.

## Supplementary Material

Supplementary Information Supplementary Tables and Supplementary References

## Figures and Tables

**Table 1:** Average values for the CO_2_/Rb, CO_2_/Ba and CO_2_/Nb ratios.

	**CO**_**2**_**/Rb**	**CO**_**2**_**/Ba**	**CO**_**2**_**/Nb**
Equatorial MIs	1105±104	97±10	557±79
Equatorial glasses	320±14	30±2	162±8
Siqueiros MIs (ref. 4)	1219±NA	100±22	239±46
Siqueiros glasses^4^	NA	68±NA	224±45
Popping Rock^2^	899±91	76±8	556±56
undersaturated ultradepleted MORB glasses^13^	1207±633	89±39	249±48
global average for MORB glasses^13^	NA	105±9	607±327

For the equatorial melt inclusions/glasses, the Siqueiros melt inclusions/glasses and the undersaturated ultradepleted MORB glasses, the 1 s.d. uncertainty is the standard deviation over the entire population of melt inclusions. For the popping rock, the uncertainty is calculated using the standard deviation over three measurements of CO_2_ content^2^ and a conservative error of 10% relative for trace elements.
