# Supplementary material for: Heterogeneity in mantle carbon content from CO2-undersaturated basalts
Source: Nat Commun. 2017 Jan 13;8:14062. doi: 10.1038/ncomms14062 (PMC5241784; doi:10.1038/ncomms14062)
Supplement: Supplementary Information — Supplementary Tables and Supplementary References [file ncomms14062-s1.pdf]

Supplementary Table 1: Long-term averages for CO_2, H_2O, F, Cl and S contents, measured repeatedly in basaltic glass standards over two years. 2SD: two times the standard deviation over the repeated measurements.

		CO_2	H_2O	F	P	S	Cl
		ppm	wt%	ppm	ppm	ppm	ppm
ALV519 (n=288)	average	165	0.17	95	302	950	53
	2SD	7	0.01	4	5	22	2
A99 (n=85)	average	1	0.14	688	2099	143	190
	2SD	2	0.01	36	71	10	12
VG-2 (n=77)	average	187	0.28	211	970	1486	259
	2SD	16	0.02	15	43	53	17
ALV981-R23 (n=37)	average	297	0.14	120	422	1081	18
	2SD	15	0.01	6	10	33	6

Supplementary Table 2: Averages of major element concentrations in standard glass VE-32 during repeated measurements during a 1-day long analytical session. The average composition of VE-32 from ref. 1 is given as a reference.

	SiO_2	TiO_2	FeO_t	MgO	Al_2O_3	CaO	Na_2O	K_2O	MnO	Cr
Gale et al. (2013)(ref 1)	51.07	1.93	11.29	6.48	14.25	10.60	2.94	0.31	0.18	NA
This study (n=19)	51.15	1.78	11.20	6.77	14.19	10.83	3.04	0.30	0.20	0.02
2SD (n=19)	0.16	0.09	0.18	0.08	0.11	0.17	0.11	0.02	0.03	0.03

Supplementary Table 3: Major element compositions of the host olivines

olivines	SiO_2	FeO	MgO	CaO	NiO	MnO	Al_2O_3	Cr_2O_3	TiO_2	Total	Mg#
E5B	40.04	12.2	47.11	0.3	0.21	0.2	0.03	0.04	0.00	100.13	0.87
E5D	39.8	13.27	46.09	0.3	0.2	0.21	0.05	0.03	0.00	99.95	0.86
E5E	39.95	11.78	47.66	0.32	0.24	0.17	0.05	0.03	0.02	100.22	0.88
E5F	40.2	11.33	47.76	0.33	0.24	0.18	0.05	0.03	0.00	100.12	0.88
E5G	40.07	11.94	47.51	0.33	0.23	0.21	0.03	0.03	0.00	100.35	0.88
E5H	40.43	10.78	48.48	0.34	0.22	0.17	0.04	0.04	0.00	100.50	0.89
E5I	40.22	11.42	47.46	0.32	0.2	0.17	0.05	0.03	0.00	99.87	0.88
E5K	40.47	9.99	49	0.3	0.32	0.16	0.06	0.04	0.00	100.34	0.90
E5L	40.17	11.59	47.47	0.33	0.23	0.2	0.08	0.05	0.01	100.13	0.88
E5M	39.89	13.74	45.67	0.3	0.17	0.2	0.03	0.01	0.02	100.03	0.86
E5N	40.31	10.97	48.28	0.3	0.29	0.16	0.04	0.02	0.00	100.37	0.89
E5O	39.89	12.59	46.81	0.29	0.12	0.21	0.03	0.02	0.00	99.96	0.87
E5P	40.08	11.93	47.45	0.33	0.25	0.18	0.04	0.03	0.00	100.29	0.88
E5Q	40.47	10.78	48.63	0.32	0.22	0.17	0.05	0.04	0.02	100.70	0.89
E5T	40.17	11.58	47.6	0.34	0.18	0.19	0.03	0.01	0.03	100.13	0.88
E5U	40.16	11.55	47.41	0.37	0.22	0.16	0.09	0.03	0.00	99.99	0.88
E5W	39.97	12.57	47.04	0.33	0.2	0.2	0.04	0.02	0.02	100.39	0.87
E5X	40.12	11.76	47.28	0.42	0.29	0.18	0.08	0.03	0.00	100.16	0.88
E5Y	40.19	11.63	47.53	0.32	0.24	0.21	0.05	0.03	0.02	100.22	0.88

Supplementary Table 4: Averages of trace element concentrations in standard glass VE32 during repeated measurements during a 1-day long analytical session. The average composition of VE32 from ref. 1 is given as a reference.

sample	Li	P_2O_5 %	K_2O %	Sc	TiO_2 %	V	Cr	MnO %	Co	Ni	Cu
Gale et al. (2013) [ref 1]			0.31		1.93			0.18			
This study (n=12)	6.15	0.19	0.29	41	1.83	340	132	0.19	42	49	61
2SD (%rel)	4.9	3.2	2.1	5.4	4.8	2.6	4.6	5.9	4.0	5.3	5.0

sample	Zn	Rb	Sr	Y	Zr	Nb	Cs	Ba	La	Ce	Pr
Gale et al. (2013)[ref. 1]		3.2	169.90	37	127	6.4	0.03	54.28	6.25	16.79	2.64
This study (n=12)	110.98	3.1	170.27	34	114	5.6	0.03	52.39	6.00	16.75	2.62
2SD (%rel)	4.4	2.7	3.0	5.1	2.9	4.1	23.5	6.8	3.2	5.4	5.3

sample	Nd	Sm	Eu	Gd	Dy	Er	Yb	Hf	Pb	Th	U
Gale et al. (2013)[ref. 1]	13.69	4.24	1.54	5.22	6.14	3.80	3.55	3.22	0.76	0.38	0.13
This study (n=12)	12.68	3.92	1.49	4.99	5.72	3.70	3.31	2.88	0.72	0.38	0.14
2SD (%rel)	6.6	6.5	7.9	6.5	4.9	6.4	8.4	10.2	7.8	8.1	11.7

Supplementary Table 5: Composition in major elements, volatile elements and trace elements of the Equatorial melt inclusions and matrix glasses, corrected for post-entrapment crystallization (PEC).

	SiO_2	TiO_2	Al_2O_3	FeO*	MnO	MgO	CaO	Na_2O	K_2O	total	%PEC
melt inclusions											
E5BMIa	51.39	0.82	15.70	8.42	0.12	8.30	13.08	2.01	0.06	100.10	0.42
E5DMIa	51.49	0.88	15.38	9.18	0.17	8.14	12.66	1.96	0.02	100.04	1.07
E5DMIb	51.12	0.85	15.20	9.38	0.14	8.35	12.87	1.93	0.04	99.98	2.43
E5EMIa	51.03	0.79	15.86	8.22	0.16	8.48	13.30	2.00	0.05	99.95	0.71
E5FMIa	50.96	0.82	15.86	7.98	0.14	8.60	13.57	1.87	0.05	100.04	0.94
E5GMIa	51.19	0.82	15.91	8.25	0.14	8.38	13.14	2.00	0.06	99.99	0.66
E5HMIb30	51.15	0.68	16.19	7.53	0.14	8.63	13.72	1.82	0.04	99.83	0.69
E5IMIa	51.25	0.92	15.82	7.93	0.14	8.43	13.49	1.84	0.05	99.76	0.81
E5JMIa	50.73	0.75	16.77	7.05	0.10	8.86	13.92	1.68	0.03	100.22	1.19
E5KMIa33	50.66	0.70	17.35	7.10	0.13	8.89	13.28	1.73	0.05	100.12	1.53
E5KMIb34	50.65	0.71	17.93	6.91	0.12	8.09	13.62	1.79	0.05	100.32	-0.31
E5LMIa	50.55	0.84	15.25	8.57	0.13	9.16	13.60	1.75	0.04	99.80	9.22
E5MMIa	51.48	1.03	15.01	9.80	0.17	8.32	11.86	2.15	0.06	99.60	0.53
E5NMIa	51.03	0.77	16.36	7.60	0.12	8.54	13.53	1.89	0.03	100.05	0.84
E5OMIa	51.15	0.94	14.98	9.07	0.14	8.74	12.94	1.88	0.05	100.05	5.78
E5PMIa	50.71	0.77	16.07	8.31	0.15	8.44	13.36	2.02	0.04	100.39	1.15
E5QMIa	51.01	0.69	16.72	7.42	0.13	8.53	13.86	1.49	0.03	100.16	0.68
E5TMIa	51.16	0.79	15.88	8.03	0.14	8.43	13.43	1.98	0.06	100.05	0.99
E5TMIb	50.74	0.77	16.34	7.71	0.12	8.17	14.18	1.80	0.05	99.77	3.03
E5UMIa	51.08	0.89	15.21	8.70	0.15	9.18	12.78	1.86	0.05	100.35	3.65
E5WMIa	52.10	0.85	16.52	7.49	0.14	7.20	13.49	2.00	0.09	99.91	-2.67
E5XMIa	51.28	1.10	14.56	9.12	0.13	9.41	12.14	2.09	0.06	99.75	2.98
E5YMIa	50.73	1.08	14.04	9.62	0.17	10.17	12.16	1.86	0.06	100.23	7.44
matrix glasses											
E5Bmx	51.56	1.03	15.07	9.21	0.16	8.19	12.49	2.08	0.07	99.85	
E5Emx	51.61	1.02	15.04	9.22	0.14	8.21	12.59	2.06	0.08	99.97	
E5Fmx	51.63	1.04	15.15	9.23	0.16	8.15	12.49	2.12	0.07	100.04	
E5Hmx	51.74	1.06	15.10	9.32	0.13	8.22	12.40	2.12	0.06	100.15	
E5Imx	51.85	1.02	15.00	9.13	0.16	8.16	12.69	2.07	0.07	100.15	
E5Kmx	51.68	1.03	15.13	9.24	0.14	8.24	12.44	2.07	0.07	100.04	
E5Mmx	51.74	1.04	15.14	9.39	0.15	8.15	12.48	2.08	0.07	100.25	
E5Nmx	51.67	1.13	15.08	9.31	0.14	8.14	12.48	2.10	0.08	100.12	
E5Pmx	51.71	1.03	15.05	9.10	0.17	8.19	12.50	2.12	0.07	99.94	
E5Qmx	51.60	1.14	15.11	9.23	0.16	8.26	12.56	2.12	0.07	100.25	
E5Wmx	51.58	1.01	15.15	9.14	0.16	8.17	12.49	2.07	0.07	99.84	
E5Xmx	51.45	0.99	15.71	8.69	0.12	8.09	12.93	2.02	0.07	100.06	
E5Ymx	51.68	1.14	14.97	9.40	0.15	7.92	12.53	2.03	0.07	99.89	

Supplementary Table 5 (continued)

	H_2O	CO	F	S	Cl	Psat	Li	P	K	Sc	Ti	V	Cr
melt inclusions													
E5BMIa	0.11	487	77.7	104	12.	1029	3.73	270	163	39.8	464	249	32
E5DMIa	0.12	295	87.9	108	6.9	633	4.34	219	75.	43.8	507	278	24
E5DMIb	0.12	287	86.5	105	6.8	615	4.43	251	68.	42.0	504	273	23
E5EMIa	0.11	432	68.8	100	11.	917	3.72	262	162	40.9	459	246	48
E5FMIa	0.14	462	73.5	983	14.	978	3.34	261	156	40.0	421	229	48
E5GMIa	0.10	402	70.1	959	10.	854	3.71	246	160	41.6	470	250	36
E5HMIb3	0.11	274	61.0	847	6.4	588	3.43	195	116	38.7	399	220	48
E5IMIa	0.12	443	70.4	103	13.	938	3.57	259	181	36.0	425	225	40
E5JMIa	0.12	239	61.3	918	5.9	514	3.11	180	85.	36.2	388	219	47
E5KMIa3	0.12	368	60.5	885	10.	785	3.48	289	138	33.3	393	225	45
E5KMIb3	0.12	384	64.3	924	11.	818	3.44	234	135	34.9	404	234	46
E5LMIa	0.11	425	72.8	964	11.	901	3.26	240	140	39.9	446	239	45
E5MMIa	0.11		89.8	105	13.		4.52	322	209	42.6	574	291	14
E5NMIa	0.12		66.5	894	9.3		3.51	190	113	38.4	399	224	46
E5OMIa	0.11	536	80.8	100	13.	1128	3.82	283	160	39.6	506	255	25
E5PMIa	0.11	415	64.8	972	9.4	881	3.41	241	160	40.3	436	245	46
E5QMIa	0.12	68	52.1	968	1.6	149	3.78	168	22.	36.7	370	238	50
E5TMIa	0.10	457	61.4	100	12.	967	3.37	250	165	42.2	481	255	32
E5TMIb	0.12	474	69.5	994	13.	1003	3.29	283	176	38.4	435	245	26
E5UMIa	0.11	378	71.3	992	11.	805	3.23	237	151	43.2	456	271	32
E5WMIa	0.11	541	79.4	106	12.	1138	4.22	296	180	42.6	525	262	33
E5XMIa	0.11	456	76.3	873	11.	965	3.55	219	129	39.4	436	242	44
E5YMIa	0.11	719	66.1	779	17.	1492	3.54	402	249	36.0	430	219	42
matrix glasses													
E5Bmx	0.12	205	106	109	20	443	4.31	350	231	41.4	572	266	40
E5Emx	0.12	193	103	105	19	416	4.41	338	220	42.5	589	275	42
E5Fmx	0.12	211	100	991	17	454	4.40	346	222	42.4	588	275	42
E5Hmx	0.12	212	100	988	17	457	4.30	340	223	43.7	594	279	43
E5Imx	0.12	191	103	103	19	412	4.51	351	221	41.8	585	273	42
E5Kmx	0.12	209	103	101	18	452	4.28	341	219	42.4	595	274	41
E5Mmx	0.12	209	103	100	17	451	4.30	352	225	41.6	592	277	42
E5Nmx	0.12	206	100	100	17	445	4.43	349	226	43.3	605	281	42
E5Pmx	0.11	206	98	975	17	443	4.14	348	228	42.5	584	269	42
E5Qmx	0.12	215	104	101	18	464	4.35	341	213	43.4	607	281	42
E5Wmx	0.12	210	99	973	17	453	4.35	342	222	41.8	591	275	41
E5Xmx	0.12	205	103	103	18	443	4.25	345	221	42.4	597	278	43
E5Ymx	0.12	203	100	100	17	438	4.35	343	208	44.7	629	290	43

Supplementary Table 5 (continued)

	Mn	Co	Ni	Cu	Zn	Rb	Sr	Y	Zr	Nb	Cs	Ba	La
melt inclusions													
E5BMIa	1160	40.	98.0	90.	72.	0.44	61.0	21.1	30.	0.845	0.0	4.55	1.0
E5DMIa	1290	40.	73.5	44.	84.	0.25	40.2	25.7	23.	0.429	0.0	2.88	0.5
E5DMIb	1240	36.	51.2	34.	79.	0.20	38.9	24.8	24.	0.384	0.0	2.82	0.5
E5EMIa	1140	40.	111	91.	69.	0.39	64.8	20.7	31.	0.739	0.0	4.91	1.0
E5FMIa	1120	38.	117	83.	71.	0.41	58.1	18.8	29.	0.823	0.0	4.44	0.9
E5GMIa	1200	38.	105	84.	59.	0.40	68.1	21.3	31.	0.872	0.0	4.53	1.0
E5HMIb3	1060	37.	125	91.	62.	0.27	69.2	18.2	24.	0.608	0.0	2.77	0.7
E5IMIa	1040	36.	90.4	78.	63.	0.43	59.4	17.9	28.	0.831	0.0	4.18	1.0
E5JMIa	951	36.	141	92.	61.	0.22	42.3	17.2	20.	0.387	0.0	2.66	0.5
E5KMIa3	949	36.	140	86.	64.	0.36	55.0	17.2	23.	0.684	0.0	4.42	0.8
E5KMIb3	977	37.	149	92.	64.	0.40	56.6	17.5	25.	0.737	0.0	4.46	0.8
E5LMIa	1010	26.	19.5	25.	65.	0.33	58.0	20.5	28.	0.784	0.0	4.00	1.0
E5MMIa	1340	43.	79.9	78.	86.	0.54	69.5	26.2	40.	1.074	0.0	5.59	1.3
E5NMIa	1020	37.	116	92.	64.	0.24	67.8	17.6	23.	0.605	0.0	2.92	0.7
E5OMIa	1170	34.	40.4	35.	77.	0.46	63.6	22.1	33.	0.987	0.0	5.02	1.2
E5PMIa	1100	39.	111	100	65.	0.35	68.5	18.6	29.	0.758	0.0	4.19	0.9
E5QMIa	1020	36.	126	86.	67.	0.05	25.8	18.6	15.	0.132	0.0	0.64	0.2
E5TMIa	1140	39.	88.9	78.	72.	0.42	64.8	20.5	32.	0.954	0.0	5.45	1.0
E5TMIb	1080	37.	75.6	62.	63.	0.44	62.9	18.2	29.	0.810	0.0	4.42	0.9
E5UMIa	1130	38.	86.6	69.	70.	0.35	71.6	21.4	33.	0.928	0.0	5.25	1.0
E5WMIa	1220	41.	89.0	87.	79.	0.49	67.9	22.7	34.	0.935	0.0	5.19	1.1
E5XMIa	1060	36.	133	73.	64.	0.41	45.5	20.7	22.	0.690	0.0	4.66	0.6
E5YMIa	980	34.	105	63.	61.	0.65	114	18.4	39.	1.308	0.0	8.08	1.7
matrix glasses													
E5Bmx	1240	43.	99.8	82.	81.	0.62	66.6	25.7	44.	1.230	0.0	6.72	1.4
E5Emx	1260	42.	102	82.	81.	0.62	70.8	25.5	45.	1.244	0.0	7.05	1.4
E5Fmx	1260	41.	99.4	81.	83.	0.65	68.8	25.7	44.	1.280	0.0	7.26	1.4
E5Hmx	1250	42.	102	83.	82.	0.65	71.2	26.4	44.	1.295	0.0	6.83	1.4
E5Imx	1250	40.	105	79.	79.	0.66	69.8	25.1	45.	1.240	0.0	6.84	1.4
E5Kmx	1230	41.	98.3	78.	82.	0.65	69.8	26.1	45.	1.240	0.0	6.84	1.4
E5Mmx	1260	41.	96.7	82.	82.	0.65	68.8	25.5	44.	1.250	0.0	6.85	1.4
E5Nmx	1250	42.	103	83.	83.	0.67	70.7	26.6	45.	1.305	0.0	7.30	1.6
E5Pmx	1220	40.	102	82.	81.	0.61	67.8	25.9	44.	1.275	0.0	6.44	1.3
E5Qmx	1250	42.	100	79.	79.	0.61	69.4	26.2	45.	1.263	0.0	6.95	1.5
E5Wmx	1220	41.	99.2	81.	81.	0.64	67.2	25.4	43.	1.208	0.0	6.49	1.5
E5Xmx	1250	41.	103	83.	81.	0.65	70.3	25.7	45.	1.288	0.0	6.97	1.5
E5Ymx	1280	42.	99.0	78.	89.	0.63	75.3	28.9	48.	1.404	0.0	7.61	1.7

Supplementary Table 5 (continued)

	Ce	Pr	Nd	Sm	Eu	Gd	Dy	Er	Yb	Hf	Pb	Th	U
melt inclusions													
E5BMIa	3.38	0.60	3.67	1.4	0.6	2.45	3.38	2.30	2.1	0.954	0.16	0.04	0.0
E5DMIa	1.98	0.45	3.16	1.6	0.7	2.79	4.04	2.85	2.8	0.853	0.08	0.02	0.0
E5DMIb	1.92	0.47	3.27	1.8	0.7	2.82	4.03	2.81	2.6	0.851	0.08	0.03	0.0
E5EMIa	3.38	0.64	3.85	1.4	0.6	2.31	3.27	2.36	2.3	0.974	0.12	0.06	0.0
E5FMIa	3.22	0.59	3.53	1.3	0.6	2.23	3.10	2.11	1.8	1.014	0.14	0.06	0.0
E5GMIa	3.34	0.61	3.65	1.5	0.6	2.40	3.32	2.26	2.5	1.029	0.15	0.06	0.0
E5HMIb3	2.51	0.46	2.87	1.4	0.5	2.01	2.94	2.00	1.7	0.756	0.11	0.03	0.0
E5IMIa	3.09	0.55	3.26	1.1	0.6	1.98	2.56	1.94	1.6	0.834	0.14	0.04	0.0
E5JMIa	1.94	0.41	2.50	1.2	0.4	1.98	2.98	1.96	1.7	0.732	0.09	0.02	0.0
E5KMIa3	2.59	0.45	3.07	1.1	0.5	1.77	2.60	1.82	1.6	0.792	0.11	0.05	0.0
E5KMIb3	2.65	0.51	3.32	1.3	0.5	1.92	2.75	1.80	1.6	0.858	0.12	0.05	0.0
E5LMIa	3.27	0.62	3.45	1.5	0.7	2.34	3.30	2.38	2.2	0.975	0.13	0.05	0.0
E5MMIa	4.25	0.75	4.67	1.9	0.8	2.87	4.44	2.98	2.7	1.207	0.18	0.07	0.0
E5NMIa	2.19	0.44	2.70	1.1	0.5	1.93	2.65	2.06	1.8	0.776	0.11	0.03	0.0
E5OMIa	3.71	0.67	4.20	1.6	0.6	2.48	3.27	2.41	2.1	1.083	0.16	0.06	0.0
E5PMIa	3.22	0.58	3.34	1.3	0.6	2.08	3.10	2.18	1.8	0.922	0.13	0.05	0.0
E5QMIa	1.23	0.27	1.99	1.1	0.4	2.09	2.90	1.96	1.8	0.602	0.05	0.01	0.0
E5TMIa	3.41	0.71	3.97	1.6	0.7	2.37	3.34	2.36	2.1	0.997	0.14	0.05	0.0
E5TMIb	2.97	0.59	3.39	1.3	0.6	2.15	2.70	1.90	1.8	0.942	0.12	0.04	0.0
E5UMIa	3.67	0.66	3.97	1.3	0.6	2.65	3.38	2.57	2.2	1.053	0.15	0.07	0.0
E5WMIa	3.81	0.70	3.93	1.6	0.7	2.71	3.64	2.49	2.1	1.117	0.14	0.05	0.0
E5XMIa	2.23	0.45	2.93	1.3	0.5	2.17	3.38	2.28	2.1	0.903	0.09	0.05	0.0
E5YMIa	4.98	0.84	4.21	1.4	0.6	2.24	3.19	2.08	1.8	1.226	0.26	0.09	0.0
matrix glasses													
E5Bmx	4.54	0.83	4.65	1.9	0.8	2.91	3.86	2.77	2.4	1.236	0.17	0.09	0.0
E5Emx	4.74	0.87	5.13	1.9	0.8	2.98	4.34	2.96	2.7	1.339	0.20	0.08	0.0
E5Fmx	4.74	0.94	4.87	1.9	0.8	3.05	4.09	2.87	2.6	1.320	0.22	0.08	0.0
E5Hmx	4.97	0.90	5.21	2.0	0.8	2.95	4.25	2.90	2.6	1.416	0.21	0.09	0.0
E5Imx	4.77	0.87	4.67	2.1	0.8	3.03	4.28	2.94	2.7	1.447	0.20	0.08	0.0
E5Kmx	4.83	0.88	5.03	2.1	0.8	2.99	4.15	2.79	2.5	1.303	0.20	0.08	0.0
E5Mmx	4.76	0.85	4.70	1.9	0.8	2.92	4.03	2.75	2.4	1.319	0.21	0.08	0.0
E5Nmx	5.01	0.95	5.21	2.0	0.8	2.86	4.32	3.09	2.6	1.392	0.20	0.08	0.0
E5Pmx	4.64	0.88	5.11	2.0	0.8	3.11	3.95	2.90	2.6	1.449	0.18	0.09	0.0
E5Qmx	4.95	0.92	4.81	2.1	0.8	2.92	4.15	3.02	2.6	1.353	0.21	0.08	0.0
E5Wmx	4.59	0.88	4.91	1.9	0.8	2.90	3.98	2.66	2.5	1.259	0.23	0.08	0.0
E5Xmx	4.88	0.87	5.24	1.9	0.8	2.95	4.19	2.89	2.6	1.379	0.20	0.09	0.0
E5Ymx	5.33	1.07	5.55	2.0	0.8	3.36	4.48	3.10	2.8	1.512	0.20	0.09	0.0

Supplementary Table 6: Raw composition in major elements, volatile elements and trace elements of the Equatorial melt inclusions and matrix glasses, uncorrected for post-entrapment crystallization (PEC).

	SiO_2	TiO	Al_2O	FeO	Mn	Mg	CaO	Na_2	K_2	total	%PE
melt inclusions											
E5BMIa	51.4	0.82	15.7	8.56	0.12	8.16	13.1	2.01	0.0	100.1	0.42
E5DMIa	51.6	0.89	15.5	9.29	0.17	7.72	12.8	1.98	0.0	100.0	1.07
E5DMIb	51.3	0.87	15.5	9.42	0.14	7.39	13.1	1.98	0.0	99.98	2.43
E5EMIa	51.0	0.79	15.9	8.33	0.16	8.16	13.4	2.01	0.0	99.95	0.71
E5FMIa	51.0	0.83	16.0	8.10	0.14	8.24	13.7	1.89	0.0	100.0	0.94
E5GMIa	51.2	0.83	16.0	8.37	0.14	8.10	13.2	2.01	0.0	99.99	0.66
E5HMIb3	51.1	0.68	16.3	7.62	0.14	8.26	13.8	1.83	0.0	99.83	0.69
E5IMIa	51.2	0.93	15.9	8.01	0.15	7.98	13.6	1.85	0.0	99.76	0.81
E5JMIa	50.9	0.76	16.9	7.16	0.11	8.48	14.0	1.70	0.0	100.2	1.19
E5KMIa3	50.8	0.71	17.6	7.18	0.13	8.33	13.4	1.76	0.0	100.1	1.53
E5KMIb3	50.7	0.71	17.8	7.08	0.12	8.37	13.5	1.78	0.0	100.3	-0.31
E5LMIa	51.5	0.92	16.8	8.14	0.15	5.32	14.9	1.93	0.0	99.80	9.22
E5MMIa	51.3	1.03	15.0	9.89	0.18	7.91	11.9	2.16	0.0	99.60	0.53
E5NMIa	51.1	0.77	16.4	7.72	0.12	8.22	13.6	1.91	0.0	100.0	0.84
E5OMIa	51.8	1.00	15.8	8.93	0.15	6.46	13.7	2.00	0.0	100.0	5.78
E5PMIa	50.9	0.78	16.2	8.46	0.16	8.15	13.5	2.05	0.0	100.3	1.15
E5QMIa	51.1	0.69	16.8	7.55	0.13	8.33	13.9	1.50	0.0	100.1	0.68
E5TMIa	51.2	0.80	16.0	8.14	0.14	8.04	13.5	2.00	0.0	100.0	0.99
E5TMIb	50.9	0.80	16.8	7.67	0.12	6.83	14.6	1.85	0.0	99.77	3.03
E5UMIa	51.6	0.93	15.7	8.75	0.15	7.88	13.2	1.93	0.0	100.3	3.65
E5WMIa	51.7	0.83	16.0	7.73	0.14	8.19	13.1	1.95	0.0	99.91	-2.67
E5XMIa	51.5	1.13	15.0	9.14	0.14	8.11	12.5	2.15	0.0	99.75	2.98
E5YMIa	51.6	1.17	15.1	9.53	0.18	7.32	13.1	2.01	0.0	100.2	7.44
matrix glasses											
E5Bmx	51.5	1.03	15.0	9.21	0.16	8.19	12.4	2.08	0.0	99.85	
E5Emx	51.6	1.02	15.0	9.22	0.14	8.21	12.5	2.06	0.0	99.97	
E5Fmx	51.6	1.04	15.1	9.23	0.16	8.15	12.4	2.12	0.0	100.0	
E5Hmx	51.7	1.06	15.1	9.32	0.13	8.22	12.4	2.12	0.0	100.1	
E5Imx	51.8	1.02	15.0	9.13	0.16	8.16	12.6	2.07	0.0	100.1	
E5Kmx	51.6	1.03	15.1	9.24	0.14	8.24	12.4	2.07	0.0	100.0	
E5Mmx	51.7	1.04	15.1	9.39	0.15	8.15	12.4	2.08	0.0	100.2	
E5Nmx	51.6	1.13	15.0	9.31	0.14	8.14	12.4	2.10	0.0	100.1	
E5Pmx	51.7	1.03	15.0	9.10	0.17	8.19	12.5	2.12	0.0	99.94	
E5Qmx	51.6	1.14	15.1	9.23	0.16	8.26	12.5	2.12	0.0	100.2	
E5Wmx	51.5	1.01	15.1	9.14	0.16	8.17	12.4	2.07	0.0	99.84	
E5Xmx	51.4	0.99	15.7	8.69	0.12	8.09	12.9	2.02	0.0	100.0	
E5Ymx	51.6	1.14	14.9	9.40	0.15	7.92	12.5	2.03	0.0	99.89	

Supplementary Table 6 (continued)

	H_2O	CO_2	F	S	Cl	Psat	Li	P	K	Sc	Ti	V	Cr
melt inclusions													
E5BMIa	0.11	489	78.11	1049	12.1	1033	3.74	271	163	39.9	4660	250	325
E5DMIa	0.12	299	88.90	1099	7.0	640	4.39	221	76.0	44.3	5130	281	247
E5DMIb	0.12	294	88.73	1083	6.9	630	4.54	257	70.4	43.0	5170	279	241
E5EMIa	0.11	435	69.30	1012	11.1	923	3.74	264	164	41.1	4620	248	488
E5FMIa	0.15	466	74.25	992	14.1	987	3.37	263	157	40.4	4250	232	488
E5GMIa	0.10	404	70.62	965	10.5	859	3.73	247	162	41.9	4730	252	362
E5HMIb30	0.11	276	61.49	853	6.4	592	3.45	196	117	39.0	4020	222	489
E5IMIa	0.12	446	71.02	1039	13.3	945	3.60	262	182	36.3	4290	227	404
E5JMIa	0.12	242	62.07	929	6.0	520	3.15	182	86.4	36.6	3920	222	483
E5KMIa33	0.12	374	61.44	899	10.8	797	3.54	294	140	33.8	3990	228	459
E5KMIb34	0.12	383	64.16	921	11.4	815	3.43	233	135	34.8	4030	233	460
E5LMIa	0.12	468	80.24	1062	12.2	989	3.59	264	154	43.9	4910	263	503
E5MMIa	0.11		90.37	1060	13.4		4.55	324	210	42.9	5770	292	141
E5NMIa	0.13		67.08	901	9.3		3.54	192	114	38.7	4020	226	473
E5OMIa	0.12	569	85.79	1069	13.8	1194	4.05	300	169	42.1	5370	271	272
E5PMIa	0.12	420	65.58	984	9.5	891	3.45	244	162	40.8	4410	248	467
E5QMIa	0.12	69	52.49	975	1.6	150	3.80	170	23.0	37.0	3730	240	504
E5TMIa	0.11	462	62.10	1012	12.9	977	3.40	252	166	42.6	4860	258	325
E5TMIb	0.12	489	71.72	1025	13.5	1033	3.39	291	182	39.6	4490	252	272
E5UMIa	0.12	392	74.06	1030	11.7	835	3.35	246	157	44.8	4730	281	335
E5WMIa	0.11	527	77.43	1039	12.6	1109	4.11	288	175	41.5	5110	255	321
E5XMIa	0.11	470	78.65	900	11.9	994	3.66	226	133	40.6	4500	250	455
E5YMIa	0.12	777	71.51	842	19.1	1604	3.82	434	269	38.9	4640	236	454
matrix glasses													
E5Bmx	0.12	205	106	1093	20	443	4.31	350	231	41.4	5720	266	409
E5Emx	0.12	193	103	1050	19	416	4.41	338	220	42.5	5890	275	427
E5Fmx	0.12	211	100	991	17	454	4.40	346	222	42.4	5880	275	423
E5Hmx	0.12	212	100	988	17	457	4.30	340	223	43.7	5940	279	435
E5Imx	0.12	191	103	1031	19	412	4.51	351	221	41.8	5850	273	423
E5Kmx	0.12	209	103	1019	18	452	4.28	341	219	42.4	5950	274	418
E5Mmx	0.12	209	103	1004	17	451	4.30	352	225	41.6	5920	277	424
E5Nmx	0.12	206	100	1002	17	445	4.43	349	226	43.3	6050	281	427
E5Pmx	0.11	206	98	975	17	443	4.14	348	228	42.5	5840	269	425
E5Qmx	0.12	215	104	1018	18	464	4.35	341	213	43.4	6070	281	426
E5Wmx	0.12	210	99	973	17	453	4.35	342	222	41.8	5910	275	416
E5Xmx	0.12	205	103	1034	18	443	4.25	345	221	42.4	5970	278	430
E5Ymx	0.12	203	100	1005	17	438	4.35	343	208	44.7	6290	290	434

Supplementary Table 6 (continued)

	Mn	Co	Ni	Cu	Zn	Rb	Sr	Y	Zr	Nb	Cs	Ba	La
melt inclusions													
E5BMIa	1170	40.5	98.4	91.0	73.2	0.445	61.3	21.2	30.6	0.849	0.01	4.57	1.07
E5DMIa	1300	40.4	74.3	44.7	85.2	0.256	40.6	26.0	23.7	0.434	0.00	2.91	0.55
E5DMIb	1270	37.1	52.5	35.4	81.0	0.212	39.9	25.4	24.9	0.393	0.01	2.89	0.53
E5EMIa	1150	41.0	112	92.6	70.4	0.402	65.2	20.8	31.4	0.744	0.00	4.95	1.02
E5FMIa	1130	38.5	118	84.3	72.1	0.416	58.6	18.9	30.1	0.831	0.00	4.48	0.98
E5GMIa	1200	39.2	106	84.8	59.5	0.408	68.6	21.4	32.1	0.878	0.01	4.56	1.01
E5HMIb30	1060	37.8	126	92.3	63.3	0.274	69.6	18.3	24.4	0.612	0.00	2.79	0.74
E5IMIa	1040	36.9	91.2	79.3	63.6	0.443	59.8	18.0	28.4	0.837	0.00	4.22	1.01
E5JMIa	962	37.1	142	93.2	62.3	0.229	42.8	17.4	20.6	0.391	0.00	2.69	0.57
E5KMIa33	964	36.7	142	87.8	65.9	0.375	55.9	17.5	24.1	0.695	0.01	4.49	0.82
E5KMIb34	974	37.8	149	92.6	64.3	0.405	56.4	17.5	25.3	0.735	0.00	4.45	0.85
E5LMIa	1110	28.7	21.4	27.8	72.4	0.371	63.9	22.6	31.6	0.864	0.01	4.40	1.17
E5MMIa	1350	43.7	80.4	78.4	86.5	0.551	69.9	26.4	40.7	1.080	0.01	5.62	1.35
E5NMIa	1030	38.1	117	93.1	64.8	0.247	68.3	17.8	24.0	0.610	0.01	2.94	0.73
E5OMIa	1240	37.1	42.9	37.4	82.2	0.494	67.5	23.4	35.4	1.047	0.01	5.33	1.28
E5PMIa	1110	39.4	113	102	66.1	0.358	69.3	18.8	30.3	0.767	0.00	4.24	0.96
E5QMIa	1030	37.1	127	87.1	68.2	0.055	25.9	18.7	15.5	0.133	0.00	0.65	0.29
E5TMIa	1150	40.1	89.8	79.1	73.6	0.432	65.5	20.7	33.1	0.964	0.01	5.50	1.09
E5TMIb	1120	38.6	78.0	64.0	65.3	0.457	64.9	18.8	30.1	0.835	0.00	4.56	0.99
E5UMIa	1170	40.0	89.9	71.9	73.2	0.365	74.3	22.2	35.2	0.964	0.00	5.45	1.09
E5WMIa	1190	40.3	86.7	85.2	77.3	0.485	66.1	22.1	33.5	0.910	0.01	5.05	1.11
E5XMIa	1090	37.8	137	75.4	66.1	0.424	46.8	21.3	22.7	0.712	0.01	4.80	0.71
E5YMIa	1059	37.6	113	68.2	66.1	0.712	123	19.9	43.2	1.413	0.01	8.73	1.86
matrix glasses													
E5Bmx	1240	43.0	99.8	82.8	81.2	0.622	66.6	25.7	44.9	1.230	0.00	6.72	1.42
E5Emx	1260	42.5	102	82.8	81.6	0.629	70.8	25.5	45.6	1.244	0.01	7.05	1.49
E5Fmx	1260	41.0	99.4	81.7	83.0	0.656	68.8	25.7	44.3	1.280	0.01	7.26	1.43
E5Hmx	1250	42.4	102	83.5	82.2	0.658	71.2	26.4	44.7	1.295	0.01	6.83	1.49
E5Imx	1250	40.4	105	79.9	79.3	0.660	69.8	25.1	45.0	1.240	0.01	6.84	1.48
E5Kmx	1230	41.8	98.3	78.5	82.6	0.652	69.8	26.1	45.2	1.240	0.01	6.84	1.47
E5Mmx	1260	41.7	96.7	82.6	82.6	0.652	68.8	25.5	44.3	1.250	0.01	6.85	1.44
E5Nmx	1250	42.0	103	83.0	83.6	0.674	70.7	26.6	45.2	1.305	0.01	7.30	1.61
E5Pmx	1220	40.9	102	82.3	81.4	0.619	67.8	25.9	44.9	1.275	0.01	6.44	1.37
E5Qmx	1250	42.3	100	79.4	79.8	0.617	69.4	26.2	45.1	1.263	0.01	6.95	1.50
E5Wmx	1220	41.1	99.2	81.3	81.7	0.646	67.2	25.4	43.7	1.208	0.01	6.49	1.50
E5Xmx	1250	41.4	103	83.0	81.4	0.653	70.3	25.7	45.7	1.288	0.01	6.97	1.55
E5Ymx	1280	42.1	99.0	78.7	89.8	0.630	75.3	28.9	48.1	1.404	0.01	7.61	1.70

Supplementary Table 6 (continued)

	Ce	Pr	Nd	Sm	Eu	Gd	Dy	Er	Yb	Hf	Pb	Th	U
melt inclusions													
E5BMIa	3.3	0.60	3.68	1.4	0.6	2.4	3.3	2.3	2.1	0.95	0.16	0.04	0.0
E5DMIa	2.0	0.46	3.20	1.6	0.7	2.8	4.0	2.8	2.8	0.86	0.08	0.02	0.0
E5DMIb	1.9	0.48	3.36	1.8	0.8	2.8	4.1	2.8	2.7	0.87	0.08	0.03	0.0
E5EMIa	3.4	0.65	3.88	1.4	0.6	2.3	3.3	2.3	2.3	0.98	0.12	0.06	0.0
E5FMIa	3.2	0.60	3.56	1.3	0.6	2.2	3.1	2.1	1.8	1.02	0.14	0.06	0.0
E5GMIa	3.3	0.62	3.67	1.5	0.6	2.4	3.3	2.2	2.5	1.03	0.15	0.06	0.0
E5HMIb3	2.5	0.46	2.89	1.4	0.5	2.0	2.9	2.0	1.7	0.76	0.11	0.03	0.0
E5IMIa	3.1	0.55	3.28	1.1	0.6	2.0	2.5	1.9	1.7	0.84	0.14	0.04	0.0
E5JMIa	1.9	0.41	2.53	1.2	0.4	2.0	3.0	1.9	1.8	0.74	0.09	0.02	0.0
E5KMIa33	2.6	0.46	3.11	1.1	0.5	1.8	2.6	1.8	1.6	0.80	0.11	0.05	0.0
E5KMIb3	2.6	0.51	3.31	1.3	0.5	1.9	2.7	1.7	1.6	0.85	0.12	0.05	0.0
E5LMIa	3.6	0.69	3.80	1.6	0.7	2.5	3.6	2.6	2.4	1.07	0.15	0.06	0.0
E5MMIa	4.2	0.75	4.69	1.9	0.8	2.8	4.4	3.0	2.7	1.21	0.18	0.07	0.0
E5NMIa	2.2	0.44	2.73	1.1	0.5	1.9	2.6	2.0	1.8	0.78	0.11	0.03	0.0
E5OMIa	3.9	0.71	4.46	1.7	0.7	2.6	3.4	2.5	2.3	1.15	0.17	0.06	0.0
E5PMIa	3.2	0.59	3.37	1.3	0.6	2.1	3.1	2.2	1.8	0.93	0.13	0.05	0.0
E5QMIa	1.2	0.28	2.00	1.2	0.4	2.1	2.9	1.9	1.8	0.60	0.06	0.01	0.0
E5TMIa	3.4	0.71	4.01	1.7	0.7	2.3	3.3	2.3	2.1	1.00	0.14	0.05	0.0
E5TMIb	3.0	0.61	3.50	1.4	0.6	2.2	2.7	1.9	1.9	0.97	0.13	0.05	0.0
E5UMIa	3.8	0.69	4.12	1.4	0.7	2.7	3.5	2.6	2.3	1.09	0.16	0.08	0.0
E5WMIa	3.7	0.68	3.83	1.6	0.7	2.6	3.5	2.4	2.1	1.08	0.14	0.05	0.0
E5XMIa	2.3	0.47	3.02	1.4	0.6	2.2	3.4	2.3	2.2	0.93	0.10	0.05	0.0
E5YMIa	5.3	0.90	4.55	1.5	0.7	2.4	3.4	2.2	2.0	1.32	0.28	0.10	0.0
matrix glasses													
E5Bmx	4.5	0.83	4.65	1.9	0.8	2.9	3.8	2.7	2.4	1.23	0.17	0.09	0.0
E5Emx	4.7	0.87	5.13	1.9	0.8	2.9	4.3	2.9	2.7	1.33	0.20	0.08	0.0
E5Fmx	4.7	0.94	4.87	1.9	0.8	3.0	4.0	2.8	2.6	1.32	0.22	0.08	0.0
E5Hmx	4.9	0.90	5.21	2.0	0.8	2.9	4.2	2.9	2.6	1.41	0.21	0.09	0.0
E5Imx	4.7	0.87	4.67	2.1	0.8	3.0	4.2	2.9	2.7	1.44	0.20	0.08	0.0
E5Kmx	4.8	0.88	5.03	2.1	0.8	2.9	4.1	2.7	2.5	1.30	0.20	0.08	0.0
E5Mmx	4.7	0.85	4.70	1.9	0.8	2.9	4.0	2.7	2.4	1.31	0.21	0.08	0.0
E5Nmx	5.0	0.95	5.21	2.0	0.8	2.8	4.3	3.0	2.6	1.39	0.20	0.08	0.0
E5Pmx	4.6	0.88	5.11	2.0	0.8	3.1	3.9	2.9	2.6	1.44	0.18	0.09	0.0
E5Qmx	4.9	0.92	4.81	2.1	0.8	2.9	4.1	3.0	2.6	1.35	0.21	0.08	0.0
E5Wmx	4.5	0.88	4.91	1.9	0.8	2.9	3.9	2.6	2.5	1.25	0.23	0.08	0.0
E5Xmx	4.8	0.87	5.24	1.9	0.8	2.9	4.1	2.8	2.6	1.37	0.20	0.09	0.0
E5Ymx	5.3	1.07	5.55	2.0	0.8	3.3	4.4	3.1	2.8	1.51	0.20	0.09	0.0

Supplementary References:

1. Gale, C.A., Dalton, C. H. Langmuir, Su, Y. & Schilling, J.-G. The mean composition of ocean ridge basalts. *Geochem. Geophys. Geosyst*. **14**, doi:10.1029/2012GC004334 (2013).
